# How to Effectively Collect and Process Network Data for Intrusion Detection?

**DOI:** 10.3390/e23111532

**Published:** 2021-11-18

**Authors:** Mikołaj Komisarek, Marek Pawlicki, Rafał Kozik, Witold Hołubowicz, Michał Choraś

**Affiliations:** 1 ITTI Sp. z o.o., Rubież 46, 61-612 Poznań, Poland or mikolaj.komisarek@pbs.edu.pl (M.K.); rafal.kozik@itti.com.pl (R.K.); 2 Institute of Telecommunications and Computer Science, Bydgoszcz University of Science and Technology, 85-796 Bydgoszcz, Poland; witold.holubowicz@pbs.edu.pl; 3 Faculty of Mathematics and Computer Science, FernUniversität in Hagen, Universitatsstrasse 11, 58097 Hagen, Germany; mchoras@itti.com.pl

**Keywords:** NetFlow, network intrusion detection, network behavior analysis, data quality, feature selection

## Abstract

The number of security breaches in the cyberspace is on the rise. This threat is met with intensive work in the intrusion detection research community. To keep the defensive mechanisms up to date and relevant, realistic network traffic datasets are needed. The use of flow-based data for machine-learning-based network intrusion detection is a promising direction for intrusion detection systems. However, many contemporary benchmark datasets do not contain features that are usable in the wild. The main contribution of this work is to cover the research gap related to identifying and investigating valuable features in the NetFlow schema that allow for effective, machine-learning-based network intrusion detection in the real world. To achieve this goal, several feature selection techniques have been applied on five flow-based network intrusion detection datasets, establishing an informative flow-based feature set. The authors’ experience with the deployment of this kind of system shows that to close the research-to-market gap, and to perform actual real-world application of machine-learning-based intrusion detection, a set of labeled data from the end-user has to be collected. This research aims at establishing the appropriate, minimal amount of data that is sufficient to effectively train machine learning algorithms in intrusion detection. The results show that a set of 10 features and a small amount of data is enough for the final model to perform very well.

## 1. Introduction

With the list of known network threats expanding every year, researchers and cybersecurity experts are constantly working on new safeguards and new tools of protection. Cybercriminals keep trying to pull newer and more sophisticated tricks to steal sensitive or personal data or cause damage to private businesses or government organizations [1,2].

To facilitate the use of machine learning to streamline network intrusion detection, good quality labeled data need to be collected. This enables the use of highly-accurate supervized learning techniques. The data-dependent algorithms are only as good as the data used to train them.

### Motivation, Methodology and Main Objectives

One of the prevailing problems of research in the domain of intrusion detection is the changing characteristics of both network traffic and the contemporary threat landscape. The pace of changes in the field is tightly connected to the intensity of the cyber-arms-race. The constant change in the threat landscape causes the benchmark datasets to lose relevance. The privacy issues and the acquisition costs make the telecom companies reluctant to provide new, labeled data, which in turn causes a constant, high demand for new, relevant intrusion detection datasets.

The motivation and contribution of this paper stems from the realization that there is a discrepancy between the datasets available to the intrusion detection researchers and the types of data that are usable in a real-world deployment of real-time ML-based NIDS. This realization comes directly from the authors’ experience in building ML-based NIDS, including the detection component for the H2020 SIMARGL project [3,4,5,6,7,8].

First and foremost, the data need to be in a flow-type format, in contrast to the packed-based IDS methods, as flow-type network characteristics offer a couple of important advantages. Flow type data can describe the activity on the network much more efficiently [9,10], and the flow-type standards are proper for high-speed networks, as the aggregations allow for significant cutback in the size of data. Additionally, the standards such as NetFlow, IPFIX or sFlow are widely adapted and recognizable in the security community [11,12]. However, not all the fields available in the IPFIX or other flow-based schemas are of value for intrusion detection. In fact, many fields do not contain any relevant information, or the information is redundant. This is reflected in the benchmark datasets, where many features have to be filtered out in the feature selection process of ML-based IDS. While feature selection is a process widely explored in the ML research community and is an important step in the formulation of any ML model, the reverse of the feature selection problem can be a major issue: not including important and informative features in the collected dataset. Having to perform feature selection is also an upfront computational cost, which has to be paid in the training phase. This is not necessary if a standard, informative set of flow-based features is established.

On top of that, many fields in the benchmark datasets are unusable from the perspective of a real-time NIDS, as they can only be calculated having collected a significant amount of flows, such as in [13]. Additionally, some widely used benchmark datasets contain custom-made features: for example, the amount of certain indicators (such as creating programs or entering certain directories on the host machine) in the actions of users in the case of benchmark datasets from the KDD family [14]. These kinds of features are realistically unobtainable in the wild for a number of reasons, starting from the inconvenience of providing these characteristics in a real-time scenario and ending with the issues of privacy.

In a real-world scenario, the deployment of an NIDS requires the collection of a sample labeled dataset from the target network due to deployment shift [15]. This is a costly and inconvenient process, so acquisition of the minimum amount of data is common sense. This paper explores the notion of minimal sets of data required for effective detection. One more important contribution stems from the fact that establishing a standardized set of features effective for NIDS helps with the use of transfer learning techniques across multiple datasets. Based on the popular flow-based data schemas, the research process presented in this paper addresses a research gap related to the verification of a list of features that contribute to network intrusion detection. In addition, the research aims to answer the question of what minimum amount of data is sufficient to effectively and efficiently train a machine learning model for threat detection. Thus, the main objectives of the paper are as follows:

1. To establish and verify an optimal set of flow-based features usable for network intrusion detection,
2. To establish the minimal amount of labeled data necessary to train a machine-learning-based NIDS for effective deployment,
3. To clear the path for anyone wishing to collect an NIDS dataset.

To validate the findings of the paper, a set of four commonly used machine learning algorithms is trained using the established flow-based features on five benchmark datasets.

To summarise and clearly state the major contributions, the paper does the following:

1. Establishes a set of effective and usable flow-based features based on five recent benchmark datasets.
2. Establishes a minimum amount of data that allows the training of an ML classifier in IDS.
3. Validates the findings by training a set of different ML models and reports the results.

**Figure 1:**
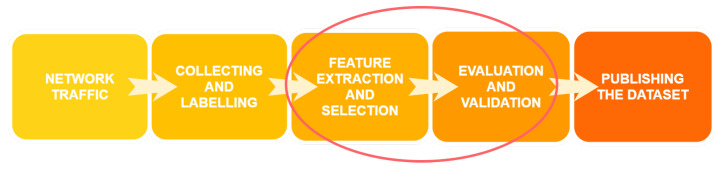
The steps required to go from network traffic to publishing of a dataset suitable for ML methods. The red ellipse indicates the focus of this paper.

As illustrated in Figure 1, five steps can be distinguished between network traffic flowing through a certain point in a network and actually publishing the dataset for the research community to use. The traffic has to be collected and reliably labeled, then an adequate and usable set of features needs to be extracted, then the collected dataset should go through evaluation and validation procedures to check its usefulness for ML procedures, and then the dataset can be published for the community to use. The work contained in this paper revolves around improving the feature extraction and selection phase, and the validation of this work is also provided.

Our previous research has focused on analyzing network traffic based on the NetFlow data format. In [3], we have proposed a new dataset derived from a real-world, in-the-wild network. The dataset is collected, described and now published. The dataset has 44 features and contains labeled data. Its use for intrusion detection has been verified and validated by using the following algorithms: random forest classifier, gradient boosting classifier, and a neural network. The detection efficiency oscillated around 99%. After the publication of the dataset, the focus of our work shifted to further improving the quality of the data collection process, for the next iteration of the dataset. This paper contains the results of this work.

The paper is structured as follows: in Section 2, a brief overview of existing approaches both in feature selection and in the way network data are prepared is presented, along with a review of articles regarding data quality. Section 3 and Section 4 describe how the datasets were collected and present the datasets used in this research article. Section 5 describes the methodology used with respect to feature selection and verification of the amount of data needed to effectively train the algorithms. Finally, Section 6 presents the results and evaluates the impact of the amount of data on the training of ML models. The paper closes with conclusions and future plans.

## 2. Related Works

In the 1990s, a group of researchers led by Professor Richard Y. Wang conducted a study in which they formulated the concept of “data quality”, which equals “fitness for use” [16]. Within this research, the definition of “data quality dimension” was also introduced, which referred to a set of attributes that defines the construct of data quality.

Since the 1990s, the evolution of the Internet has caused the approach to the field of data quality to change dramatically. Li Cai and Yangyong Zhu present the current challenges in the era of big data in their research [17]. They identified several problems that arise in this day and age, the first being the variety and complexity of data sources and types. This phenomenon appears in the literature, e.g., in [18] or [19]. Another challenge is the huge amount of data coming from all directions and the fact that it is difficult to assess the quality of these data in a short period of time. Moreover, the change and validity of these data is very short, which makes data processing even more important nowadays. In the rest of the paper, the authors proposed the establishment and a hierarchical structure of a data quality framework and presented a process for assessing the quality of large amounts of data.

The authors of [20] provide an extensive analysis and review of currently existing data quality approaches to big data. At the very beginning, they cite the concepts of big data and present the life cycle of such data. The initial phase of collection starts with data generation or retrieval, then one enters the phase of data acquisition to move on to data storage in the next stage and then to data processing and analytics. The whole process ends with the data visualization aspect. In the next part of the article, the authors analyze the quality of data by presenting a wide range of articles that show the problems of maintaining quality in large datasets. They conclude that there is no complete reference model for data quality and management in big data.

In addition to the quality of the data itself, the size of the dataset has quite a significant impact on the machine learning process. The authors of the research paper [21], who base their research on the medical domain, examine the impact of the size of the learning set on model quality and performance. For this purpose, they conducted a set of experiments on six popular machine learning models using medical datasets. To measure the effect of data volume on model performance, they prepared a set of three subsets of different sizes and a series of metrics to compare performance. The authors of the paper emphasize that their study shows that it is not the size that affects the performance of the classifier but the degree of the dataset that represents the original distribution. Another conclusion from the research is that for a limited dataset, the AdaBoost and Naïve Bayes classifiers perform best and the decision tree classifier performs worst.

The authors of [22] used three distributed algorithms—extreme learning machines (ELM), distributed random forest, and distributed random boosted-trees—to detect botnet attacks. In the research paper, they presented the concepts and architecture of the system, which was based on big query data processing. Network data analysis in the form of NetFlow was used as a use case. The results provided in the conclusion show that this is a very promising work.

In [23], the authors have addressed the topic of data characterization, namely the problem associated with the imbalance of infected samples from normal traffic. For this reason, the authors presented a number of studies regarding data balancing and its impact on various machine learning algorithms.

The authors of [11] analyze data in the form of NetFlow and IPFIX with respect to network traffic monitoring. They point out at the very beginning of the paper that these protocols are used for scaled fast network flow export. The article itself introduces the reader to the history of these two protocols, and outlines the fundamental differences between them. An example architecture of a flow monitoring system is presented by the authors. In addition, a comprehensive comparison of network traffic collection tools is provided.

A similar analysis is performed by the authors of [24]. They rely on a review of machine learning and data mining methods used in cyber analytics to support intrusion detection.

The effect of data volume on machine learning effectiveness has also been examined in [25], in which researchers use data from Tweets to test algorithms such as decision trees, naïve Bayes, nearest neighbor and radial basis function network. Based on the results, the developers suggest that increasing the data size improves performance but the effect of this improvement decreases as the sizes of the datasets increase. They also note that it is more important to add additional samples to small datasets than to larger ones. The best classifier proved to be naïve Bayes, which was also the fastest in the training process and achieved good results on the smallest datasets.

A slightly different area is explored by the authors of [26]. They focus on investigating the optimal size of the number of features in the random forest algorithm. The authors’ conclusions emphasize that the hypothesis is true and that there is no functional relationship between the optimal size and the characteristics of the datasets being checked. They confirm this after using the out-of-bag error method and SearchSize using random forest.

This work is mainly concerned with good quality network data based on NetFlow and selecting the optimal number of features and set size with respect to intrusion detection. There are many research articles that base the detection of undesirable events in computer networks by verifying NetFlow data.

In [27], the authors use NetFlow-based network traffic. K-means and genetic algorithms were compared and tested. These two approaches were used to find the undesirable parts of the network traffic. Ultimately, the results that were obtained in this paper prove that the genetic algorithm is better suited to calculate the so-called survival curves.

The authors of [28] perform intrusion detection in network traffic. However, the proposed method is based on identifying anomalous end-user nodes and their network traffic patterns. The authors point out that frequently changing IP addresses make their method ineffective. It can be concluded that when identifying anomalies, IP addresses and ports should not be used.

The network traffic intrusion detection architecture proposed in [29] is based on the use of a time series clustering algorithm. The authors show that the algorithm is able to detect anomalies in live data without any prior knowledge of the data.

In [4,5], the authors focused on presenting the concept of architecture and software, the task of which was to analyze the traffic in real-time from the data provided by the stream. The authors use the scalable Apache Kafka environment, Apache Spark and the Elasticsearch database for this purpose. For efficient intrusion detection, network flows in the data stream are grouped by source IP address into one-minute time windows.

The authors of [30] propose a moderate architecture of a convolutional neural network (CNN) to facilitate a decrease in the resources consumed by computations in large-scale intrusion samples, attempting to better the classification metrics.

In [31], the researchers present two approaches grounded in wavelets to effectively mine and analyze network security log databases. Using wavelets allows the extraction of adequate frequency components. The authors conclude that using wavelet transforms grants the ability to de-noise the data, which in turn permits faster querying.

The authors of [32] introduce multiscale Hebbian learning to tackle the challenge of inadequately labeled data in network intrusion detection. The experiment conducted on the UNSW-NB15 dataset shows that the approach can spot overlapping classification boundaries.

A mixed wavelet-based neural network model for cyber security situation prediction is evaluated in [33]. The approach shows significant improvements over the state-of-the-art.

In [34], a comprehensive survey of machine learning and deep learning approaches to intrusion detection can be found. The study taxonomizes the IDS systems by detection method and source of data, then lists the common learning algorithms employed for IDS, including both the shallow and deep learning models.

## 3. Machine Learning over NetFlow Data

In this work, the main focus of the research is on finding a suitable data scheme to detect intrusions in network traffic in an effective manner. This paper showcases the process of evaluating flow-based features in the task of network intrusion detection. This work also establishes the minimum amount of data needed to train the various machine learning algorithms.

The most popular versions of NetFlow are versions 5 and 9, thus this work will focus on fields obtainable using this format. The topic of network intrusion detection with the use of NetFlow is a well established approach in the research community. Multiple papers have been published proving that it is possible to build a working ML IDS based on flow-based data [35].

### 3.1. Collecting Data

Data collection is of crucial importance in the entire process of building a machine learning model. Providing good quality and sufficient amount of training data allows the algorithm to be trained effectively. NetFlow collection can be achieved by tools such as Nprobe or Ntop [36]. The ability to collect traffic is also possible using a configured ElasticStack environment. Nprobe [37] is a tool that includes both a probe and a collector. It grants the ability to export traffic as NetFlow v5/v9/IPFIX. The authors of this tool point out the following as the main advantages of this software: the full support for IPv4 and IPv6, and the ability to automatically export to an SQL database and to a Kafka stream, as well as low CPU and RAM resource consumption.

Nowadays, with the size of network traffic growing at a staggering pace, there is a need for scalable solutions that can handle the increasing amount of data. In [5], an in-depth description of the software and its architecture is presented. The detector relies on collecting data using a NetFlow collector, and the data are transferred directly to the Kafka stream. The data are then processed by the solution, which performs intrusion detection, and the result along with the features of the sample are recorded to the Elasticsearch database. This solution architecture is highly scalable and tools such as Apache Kafka guarantee redundancy.

### 3.2. Datasets

Five datasets were selected for testing on NetFlow-based collections. They represent recent network traffic and therefore they reflect relatively current network behavior. The authors of [38] converted five popular datasets to a strict NetFlow format. A detailed description of the individual features available within the sets can be found in Table 1. The datasets provide 33 numeric parameters and four text parameters.

- UNSW-NB15 [39]—The dataset was created in 2015, with the IXIA PerfectStorm tool. Using this software, clean traffic and various types of network anomalies were generated. Approximately 100 GB of data stored as PCAP files was collected and thanks to the developers at The Cyber Range Lab of the Australian Centre for Cyber Security (ACCS), the collection has been made public as part of further research into improving network security. The structure of the collection originally contained 49 features and encompassed 2,218,761 samples of clean traffic, which is about 87.35% of the whole collection. The rest, i.e., 321,283 network frames, is made up of executed attacks.
- BoT-IoT [40]—Developers in Australia (ACCS) also created this dataset in 2018. In this case, a network flow taking place in a real network environment was recorded. This collection estimates about 69 GB of data in PCAP format and contains 42 features. The diversity of traffic in this collection is very uneven as it contains only 477 frames and there are 3,668,045 flows of the infected traffic. This results in normal traffic of only 0.01%.
- ToN-IoT [41]—this data collection is very similar to the BoT-IoT collection, as it also contains very many attacks and very little normal traffic. The collection comes from the IoT network, more precisely from service telemetry data, and was recorded in 2020. The number of infected frames equals 21,542,641 samples while normal traffic is only 796,380 flows. This represents a percentage of 96.44% for the infected samples and 3.56% for normal traffic, respectively.
- CSE-CIC-IDS2018 [42]—in 2018, another dataset made available through a collaboration between two organizations: Communications Security Establishment (CSE) and the Canadian Institute for Cybersecurity (CIC), was released. This is a very realistic set, as the scenario was designed using the infrastructure of five large organizations and server rooms. Normal traffic was generated by human users and several different machines were used to attack these networks. The whole collection contains 73 features and consists of a large amount of data amounting to 16,232,943 flows. The attacks in this collection represent 2,748,235 samples and the normal traffic represents 13,484,708 flows.
- UQ-NIDS [43]—a dataset that was created by combining the four previously presented datasets. It represents the advantages of shared datasets, where it is possible to combine multiple smaller datasets, which leads to a larger and more versatile NIDS dataset containing flows from multiple network configurations and different attack settings. This network dataset contains 11,994,893 flows, of which 9,208,048 (76.77%) are benign flows and 2,786,845 (23.23%) are attacks.

### 3.3. Feature Selection

The first stage of the research will be to sift and select features from the NetFlow datasets and thus establish the most relevant flow-based set of features to feed the ML algorithms. Only those features will be extracted that show the best performance in the intrusion detection process and positively affect the detection metrics of the model. Several selection techniques will be applied to maintain the validity results and provide the most relevant set of features.

In a 2020 survey of feature selection techniques [44], the authors describe the different variants and possibilities to correctly pick features. The authors of [44,45] indicate that feature reduction leads to reduced complexity, which translates into reduced computation time. The feature selection techniques that will be used in the following experiments can be divided into four categories [46,47,48]:

- Filter methods,
- Wrapper methods,
- Embedded methods,
- Hybrid methods.

The categorical values that are present in the datasets to be subjected to the feature selection technique must be preprocessed by functions such as ordinal encoder, one hot encoder, or other methods [49]. The list of features found in the five analyzed datasets in this paper has several categorical values, such as IP addresses or destination and source ports. These features are not involved in the research conducted in this article and are omitted, so only the non-categorical values remain in the dataset, although an overview of all methodologies that can be used when categorical values are present in the data can be found in [50]. The rejection of these features is dictated by the fact that IP addresses and ports change dynamically from network to network and relying on them limits the capabilities of the algorithm.

The first technique that was used to study the number and effectiveness of features is LASSO regularization [51,52], which is an embedded method and combines both wrapping and filtering methods. The term regularization refers to the concept the application of which is to prevent data overfitting. The way this method works is based on adding penalties to the parameters to reduce the freedom of the models. There are two main regularization techniques, namely ridge regression and LASSO regression [52]. In the LASSO technique, shrinkage is used. This is where the data values are shrunk towards the center point as the mean. The LASSO regularization technique itself seeks to create simple models with a reduced list of parameters. This type of regression is used for models that exhibit high levels of collinearity, or to automate variable selection/parameter elimination. When the model uses an L1 regularization technique then it is called LASSO regression. If it uses an L2 regularization technique, then it is called ridge regression. Due to the fact that this paper will use the L1 approach, a penalty equal to the absolute value of the coefficient size is added in this technique.

The choice of the L1 technique in this research was dictated by a key difference between the L1 and L2 techniques. Namely, LASSO reduces the coefficient of a less important feature to zero, completely eliminates it from the dataset, which works perfectly for feature selection. In turn, L2 is mainly used to avoid the over-fitting problem.

Some coefficients may become zero and be eliminated from the model. Larger penalties result in coefficient values closer to zero (ideal for creating simpler models). Because it is a linear model type, a penalty is imposed on the coefficient that participates in the multiplication of each predictor. The mathematical notation of this technique is shown below:

(1) $$\frac{1}{2n}\sum_{i=1}^{n}{\left(y_{real}-y_{pred}\right)}^{2}+\alpha \sum_{j=1}^{p}|a_{j}|$$

In Equation (1), it is assumed that the dataset has *n* instances and *p* features. The $y_{real}$ and $y_{pred}$ parameters define for us the predicted value result and the real value result. The parameter *a* is considered a hyperparameter in this formula. The purpose of regression is to reduce the values of the coefficients to exclude useless features. When *a* is 0, it reverts to the original linear regression. If α is too large, it neglects the first part of the cost function and the results are unreliable. The LASSO regression concept ultimately leads to the optimization of the cost function. This is achieved by reducing the absolute value of the coefficients. This technique is only likely to work if the features are normalized.

Random Forest Importance [53] is another method that was used to compare the effect of parameters on model quality. Calculating the importance of features found in a dataset using the random forest technique answers the question of what features will be appropriate in subsequent classification, as well as regression. In his 2001 paper, Breiman [54] presented a technique called out-of-bag (OOB) importance score. Its operation is based on calculating the difference between the original mean error and the randomly permuted mean error in the OOB samples. All feature values are stochastically changed for each tree and using this operation using random forest model to predict this permuted feature and obtain the mean error. If the average error decreases drastically, it means that the feature is strongly correlated [55].

Another way that shows the importance of a feature is to rank the tree due to the decrease in impurity (Gini impurity) relative to all trees [56]. According to the principle of the algorithm, the most impure trees are at the beginning and the least impure trees are at the end. With this division, a set of most important features can be easily created. The mathematical formulation of this procedure can be represented as follows. For each node *A* for the decision tree, the partitioning is performed after decreasing the impurity of the node *R*(*A*). The impurity of a node is represented by a Gini index. The determination of the Gini Index can be defined as subtracting the sum of squared probabilities of each class from one. If the samples of class Z are contained in a subset of A then the impurity Gini(A) can be defined as seen in Equation (2)

(2) $$R\left(Z\right)=1-\sum_{j=1}^{Z}{\left(P_{j}\right)}^{2}$$

where $P_{j}$ is the relative frequency of class *j* in *Z*, or in other words, it is the probability of an element being classified in another class. After splitting Z into two different nodes *Z*_1_ and *Z*_2_ with two different data sizes *N*_1_ and *N*_2_, the Gini index can be defined with the formula in Equation (3):

(3) $$Gini_{split}\left(Z\right)=\frac{N_{1}}{N}Gini\left(Z_{1}\right)+\frac{N_{2}}{N}Gini\left(Z_{2}\right)$$

The splitting of a given node occurs when $Gini_{split}\left(z\right)$ is the smallest value. The importance value of feature $X_{j}$ in a single tree $T_{k}$ is defined by the formula in Equation (4):

(4) $$S_{k}\left(X_{j}\right)=\sum_{t\in T_{k}}\Delta Gini_{split}\left(t\right)$$

Finally, this formula is applied to every tree in the set. In the above Equation (4), *t* represents a single node belonging to a single tree $T_{k}$, for each node, the Gini split method is calculated. This is how the best influencing features on the model are selected using the RF importance method.

The final feature selection method used in this paper is the chi-square method [57]. It is based on the calculation of chi-square between each feature and the target value.

(5) $$chi^{2}=\sum_{e_{t}\in \left\{0,1\right\}}\sum_{e_{c}\in \left\{0,1\right\}}\frac{{\left(N_{e_{t}e_{c}}-E_{e_{t}e_{c}}\right)}^{2}}{E_{e_{t}e_{c}}}$$

In Equation (5) *N* is the observed value of w, and *E* the expected value. $e_{t}$ takes the value of 1 if the document contains the term *t*, and 0 otherwise. $e_{c}$ takes the value 1 if the document belongs to class *c*, and 0 otherwise.

### 3.4. The Effect of Training Data Size on the Model

As part of the development of the area of network traffic intrusion detection, the next objective of this paper was to conduct a study related to finding a sufficient amount of learning data that can be used to effectively teach machine learning or neural network algorithms, taking into account the aspect of no loss of efficiency of a given algorithm. The process of testing such an assumption started by dividing the sets into smaller parts. Given five different datasets based on the same scheme, nine subsets containing different numbers of samples were extracted from each of them. To maintain the greatest reliability between data iterations of testing within each larger subset, data from smaller subsets were included. The process of the algorithm was shown in the pseudocode format in Algorithm 1. The preparation of this stage of the research began with loading all five sets into memory, then within each iteration/set the nine smaller subsets were extracted. For each subset, four algorithms were trained and their performance was tested on the test data by generating ROC curve plots and a set of metrics. The ROC curve is a graphical representation on a graph on the Y axis of a value relating to specificity, and on the X axis of a value relating to a false-positive rate. The range of values of this curve is represented from 0 to 1. Values closer to 1 indicate better performance.
**Algorithm 1** The process of extracting and splitting a dataset.**Require:**datasets[D1,D2,D3,D4,D5] trainDataset,testDataset=dividing_the_set_in_a_ratio_of_70_to_30 subDataset=[10,100,500,1000,2000,10000,20000,30000,N] tempDataset=[] **for each** d∈trainDataset **do**  **for each** size∈subDataset **do**   tempDataset=d[size]+tempDataset   d=d−tempDataset  **end for** **end for**

The final phase of testing was the preparation of a summary set, which contained a set of metrics with respect to the trained model. In this phase, the standard split of training to test data was 70 to 30. Several metrics were used to evaluate the performance and correctness of the model to give a good summary of the results. The models were evaluated [58,59] using: Accuracy (ACC-Equation: (6)), Precision (Pr-Equation: (7)), Recall (Re-Equation: (8)), F1-Score (Equation: (9)), Balanced accuracy (BCC-Equation: (10)) [60] and the Matthews correlation coefficient (MCC-Equation: (11)) [61]. Within the listed metrics that describe the performance of given algorithms in this study, values such as True Positive (*TP*), True Negative (*TN*), False Positive (*FP*), False Negative (*FN*) were needed to calculate them.

Presented below are the individual formulas that were considered in the process of evaluating the performance of the algorithm.

(6) $$Accuracy=\frac{TP+TN}{TP+FP+FN+TN}$$

(7) $$Precision=\frac{TP}{TP+FP}$$

(8) $$Recall=\frac{TP}{TP+FN}$$

(9) $$F1=2*\frac{Recall*Precision}{Recall+Precision}$$

(10) $$BCC=\frac{\frac{TP}{TP+FN}+\frac{TN}{TN+FP}}{2}$$

(11) $$MCC=\frac{TN*TP-FN*FP}{\sqrt{\left(TP+FP)(TP+FN)(TN+FP)(TN+FN\right)}}$$

### 3.5. Classification Models

To validate the effectiveness of the established set of features, four different widely used ML algorithms were trained and tested. The algorithms were chosen to cover different paradigms of machine learning: AdaBoost for tree-based based algorithms, ANN for gradient-based algorithms, naïve Bayes for Bayes theorem-based algorithms. The random forest was included, as in our experience it is a good fit for flow-based IDS. The performance of the four algorithms was tested for optimal dataset size and network traffic intrusion detection.

The first reported algorithm in the set is random forest. As the name suggests, this algorithm consists of multiple decision trees that form a forest. This tree-based algorithm has been utilized in many IDS and research papers; some examples of its use and effectiveness can be found, e.g., in [62,63,64]. The model is trained using bagging (bootstrap aggregation) techniques. The outcome of the algorithm is determined by the average scores from each tree. The random forest eliminates the limitations of the decision tree algorithm. It reduces the overfitting to the datasets and increases precision [65]. Entropy is worth mentioning within this algorithm. Entropy [66,67,68] is a measure of disorder or, in other words, uncertainty. It is expressed by the Formula (12):

(12) $$E\left(x\right)=\sum_{i=1}^{c}-p\left(x_{i}\right)\log_{2}p\left(x_{i}\right)$$

In this formula, $p(x_{i})$ expresses the measure of the probability of the frequency of the occurrence of element/class “*i*” in the data. When using machine learning algorithms, the goal of the data scientist is to reduce the disorder. The metric for reducing this disorder is expressed by the following Formula (13):

(13) IG(Y,X)=E(Y)−E(Y|X)

The operation of this metric is to subtract the entropy of *Y* from a given *X* from the entropy of *Y* itself, given the additional information X has about Y. This process is called information amplification. As there is more uncertainty reduction, more information about *Y* is obtained from *X*. Entropy as a metric is involved in the decision tree process. During the construction of decision trees, data partitioning is calculated using information gain (IG). IG is a measure that defines how much “information” a feature gives us about a class. The attribute with the highest information gain will be split first in the tree construction process. The number of trees hyperparameter was set to 100.

The second algorithm used for intrusion detection in this paper is the adaptive boosting classifier (AdaBoost). The algorithm itself has its effectiveness proven in the scientific literature on intrusion detection [69,70,71]. The technique involves adding an element of boosting and adaptively adjusting the weights when a misclassification is made. In this process, weak weights are converted to strong weights. Boosting is used to reduce bias as well as variance for supervized learning. The algorithm used in this research had the learning rate set to 1 and the N_components hyperparameter to 100.

The third model used in the intrusion detection study was based on a neural network. A simple neural network was designed, which consisted of two hidden layers and two dropout layers. The first hidden layer contains 32 neurons and the second layer contains 16 neurons, the two abandonment layers were set to 0.01, while the activation layer in the hidden layers was set to the Rectified Linear Unit (ReLU). The last layer contained the number of neurons equal to the number of classes and used a softmax activation function. The loss function was set to the “categorical_crossentropy” method while the chosen optimization algorithm was adaptive momentum [72]. Early stopping stopped the learning process at 16 epochs, and the batch size was set to 20.

The final algorithm used in our study is naïve Bayes. This classifier is based on Bayes’ Theorem with the assumption of independence of predictors. It states that the presence of a feature in a class is not dependent on any other feature. The naïve Bayes model is easy to build and is particularly useful for very large datasets [73]. The default hyperparameters were used for this classifier.

All types of models that have been selected for this research have characteristics that make them promising for an in-the-wild application of IDS. The artificial neural network model was used because of the fact that ANNs continue to learn even when other methods reach their full potential. Using more data for training this algorithm can improve the detection performance results. While there are a myriad of ANN-based algorithms that could be applied here—convolutional neural networks, recurrent neural networks, etc.—there already exists plenty of research that deals with the specifics of certain deep neural network paradigms in IDS. The focus of the use of ANNs in this paper is only on validation of the feature set and the minimal amount of data. The second model to be discussed is the AdaBoost. This algorithm is fast, easy to use and does not require extensive tuning of hyperparameters. Random forest has proven itself in many network attack studies and its performance has always been high and the results satisfactory; the authors found promising results of using this algorithm in previous work [3,4,5]. The last chosen algorithm is the naïve Bayes classifier. Its performance is based on a strong assumption of independence, and in a literal sense, it refers to the statement that the probability of one attribute does not affect the probability of another. One of the most important aspects of why this algorithm was selected for validation of this research is that it can perform better than other algorithms in situations with little training data.

In summary, parameter tuning was applied for each model to obtain the best possible results. For this purpose, the GridSearch technique was used. The effects of parameter tuning for each model can be observed in Table 2. The table contains two columns that represent the name of the parameter and its final value. A 10-fold cross validation was used during the GridSearch process.

## 4. Experiments and Results

**Figure 2:**
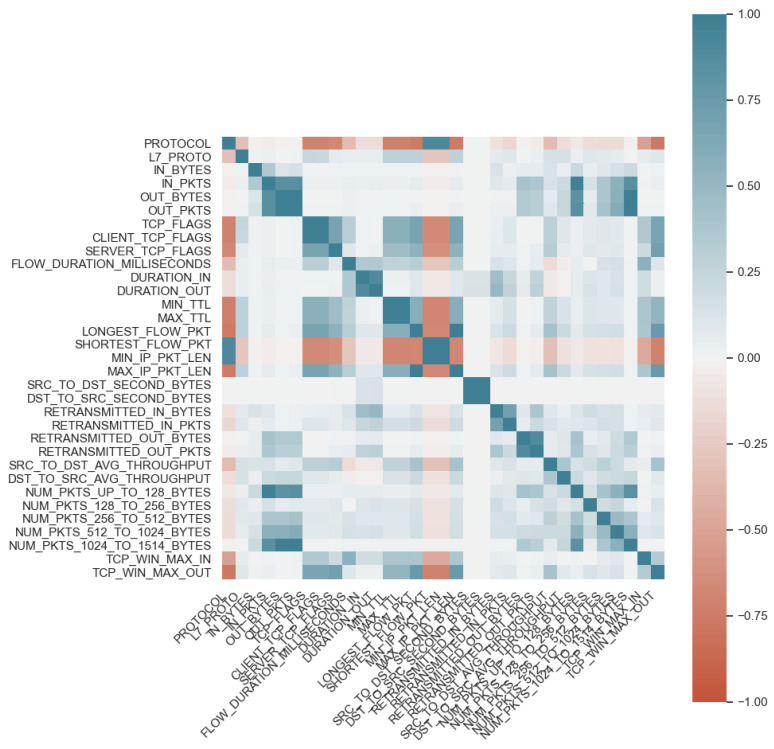
The correlations of features in the datasets: NF-UNSW-NB15, NF-BoT-IoT, NF-CSE-CIC-IDS2018, NF-UQ-NIDS, NF-ToN-IoT.

The first stage of research in this paper was to select an appropriate number of features from five network datasets based on the NetFlow format. Each set was subjected to a feature selection process. The correlations of features with each other in the dataset were evaluated. The following values were eliminated from the set due to high correlation with other features: OUT_PKTS, CLIENT_TCP_FLAGS, MIN_TTL, SHORTEST_FLOW_PKT, DST_TO_SRC_SECOND_BYTES. A summary of the exploration of the feature space offered by the NetFlow schema culminates in Figure 2, showing the feature correlation map.

**Figure 3:**
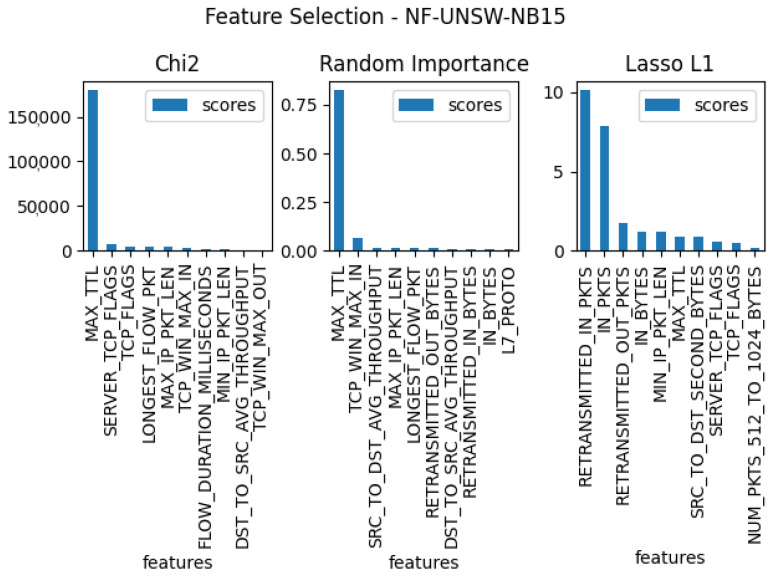
The top 10 best features determined by using three algorithms: LASSO L1, random forest Importance, and Chi2 for dataset nf-unsw-nb15.

**Figure 4:**
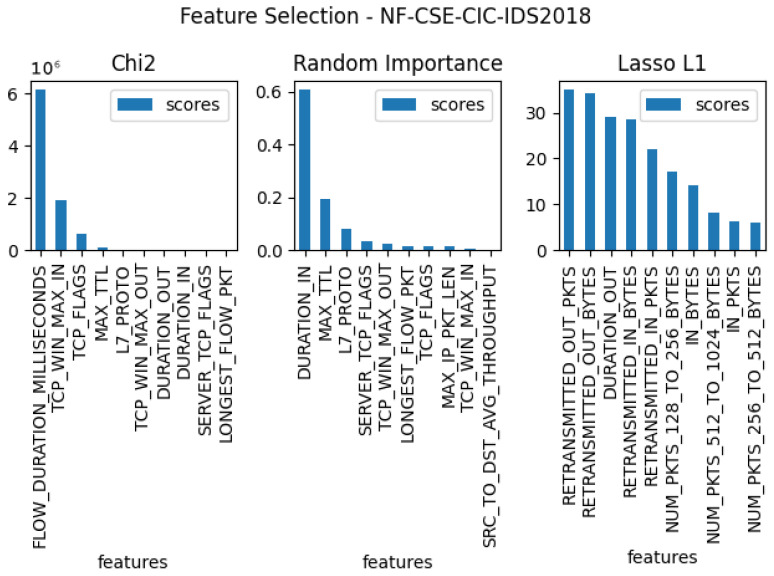
The top 10 best features determined by using three algorithms: LASSO L1, random forest importance, and Chi2 for dataset nf-cse-cic-ids.

**Figure 5:**
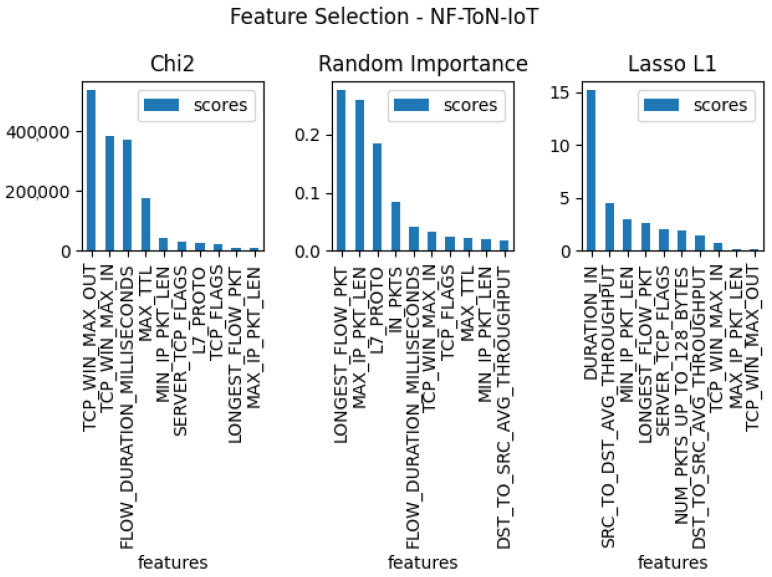
The top 10 best features determined by using three algorithms: LASSO L1, random forest importance, and Chi2 for dataset nf-ton-iot.

**Figure 6:**
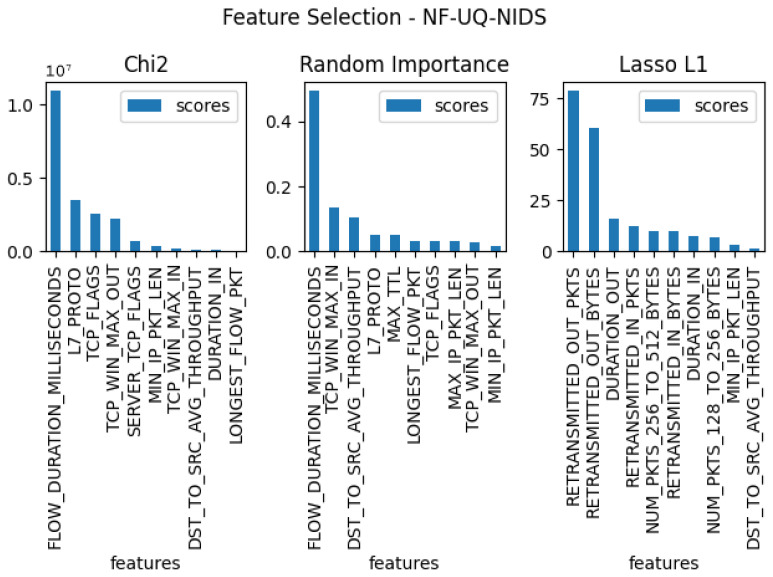
The top 10 best features determined by using three algorithms: LASSO L1, random forest importance, and Chi2 for dataset nf-uq-nids.

**Figure 7:**
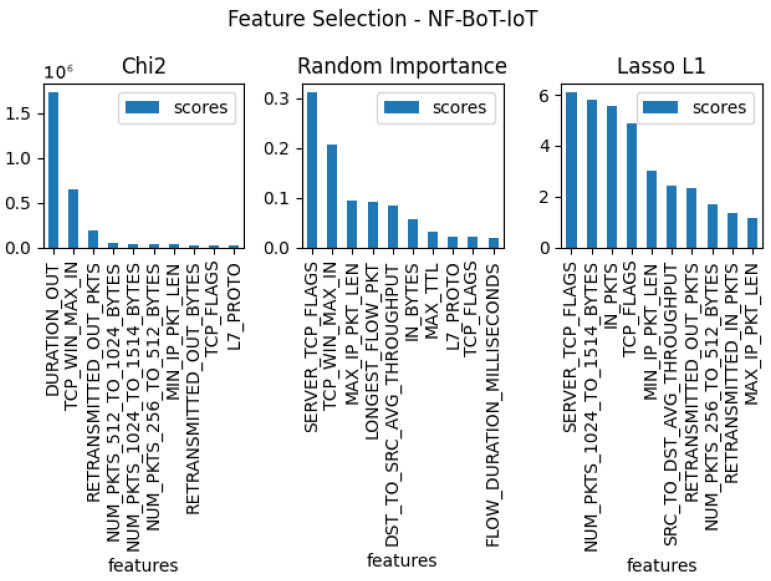
The top 10 best features determined by using three algorithms: LASSO L1, random forest importance, and Chi2 for dataset nf-bot-iot.

In the next stage of the testing, three separate feature verifications were conducted for each of the five sets, with the goal of selecting the 10 features that were most useful for the entire set. For this purpose, the methods used for the tests were: Chi2, random forest importance, and LASSO L1. The results of these tests for each dataset can be found in Figure 3, Figure 4, Figure 5, Figure 6 and Figure 7. From the above results of examining the ability of individual parameters, it can be clearly concluded that the set of features worth considering is not 33. To achieve similar results with such a list of anomalies, it is sufficient to select 10 features.

**Figure 8:**
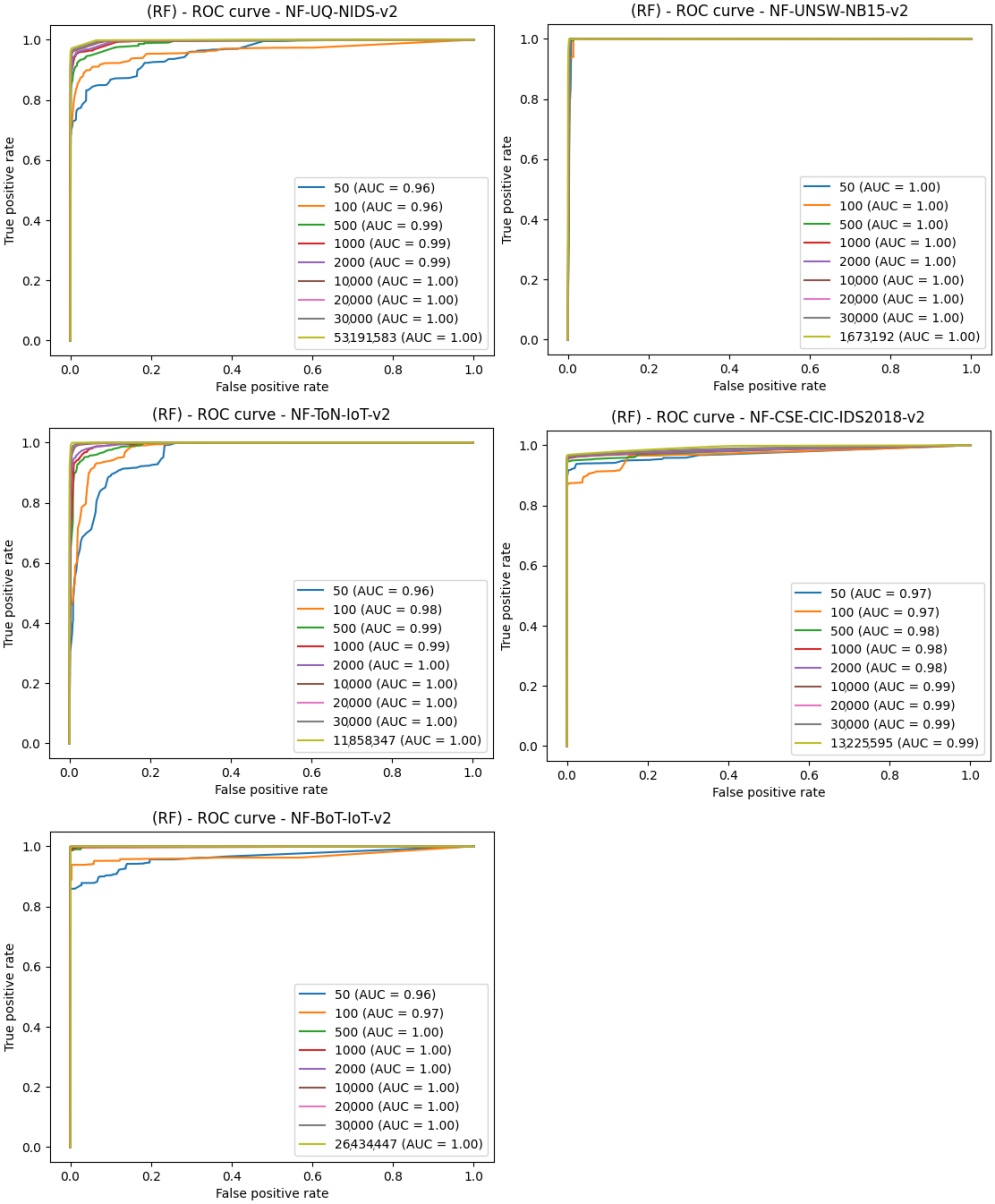
Comparison of the number of samples and machine learning effects—ROC curve plot for the random forest algorithm.

The second stage of the research was conducted to verify the effect of the data volume on intrusion detection in network traffic. Using five NetFlow datasets further divided into subsets with appropriate number of samples, the results in the form of a ROC curve plot were collected. Nine smaller subsets were separated from each of the five sets. The sizes of these subsets were as follows: 50, 100, 500, 1000, 2000, 10,000, 20,000, 30,000, and the last subset was equal to 30% of each total dataset. Another important note is that 50% of each subset consists of infected traffic, and 50% of clean traffic. The first algorithm that was trained on all the subset datasets used in this paper was random forest. The configuration of this model was default, so the parameter selection was not modified. The result for all the datasets and the random forest algorithm is shown in Figure 8.

**Figure 9:**
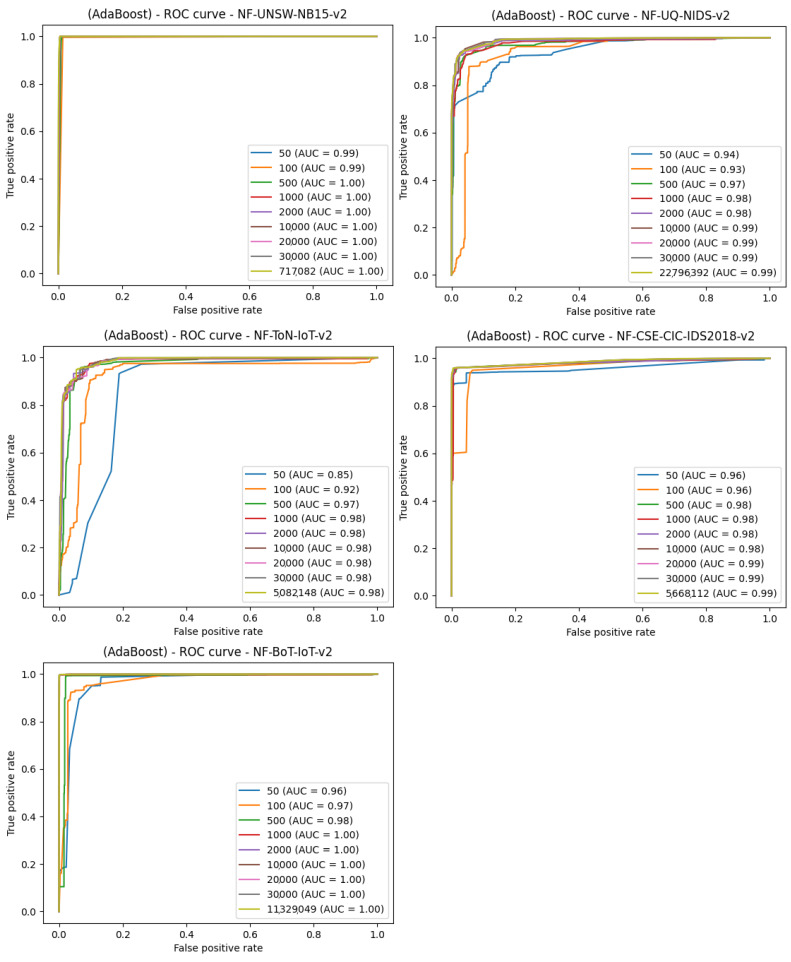
Comparison of the number of samples and machine learning effects—ROC curve plot for the AdaBoost algorithm.

It appears that for such anomalies, it only takes about 500 samples, i.e., 250 attack and 250 benign traffic samples, to perform detection with the effectiveness similar to much larger subsets. The second algorithm used to study the size of data needed for learning is AdaBoost. The data splitting is identical to the previous algorithm and the results for all the datasets and the AdaBoost algorithm have been shown in Figure 9.

**Figure 10:**
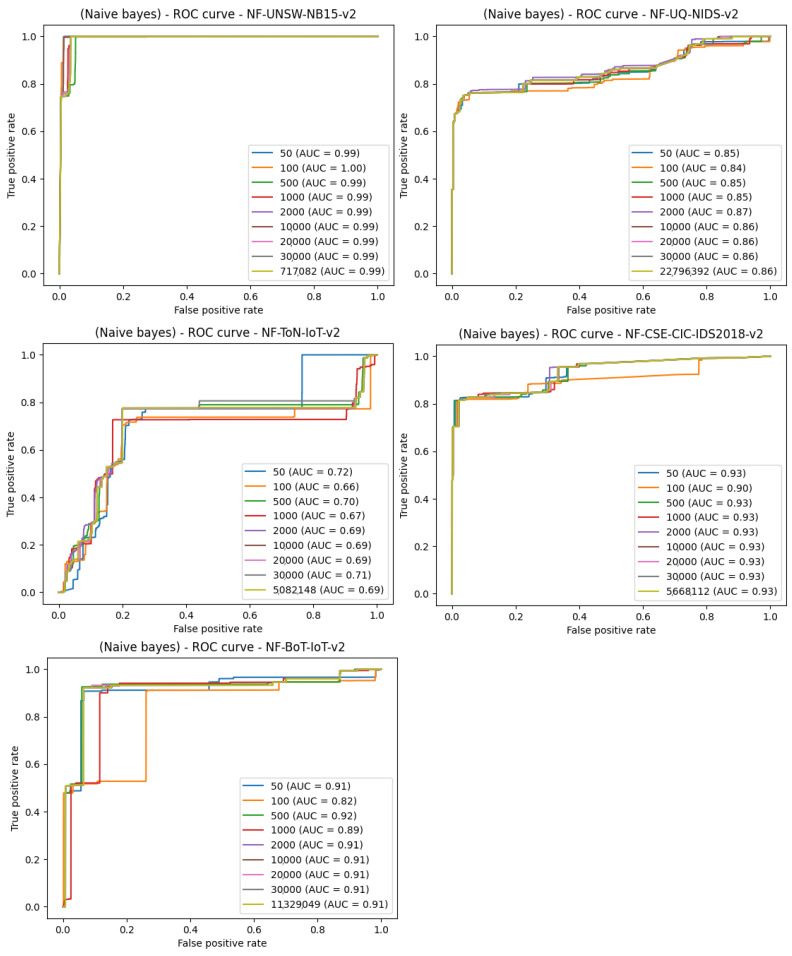
Comparison of the number of samples and machine learning effects—ROC curve plot for the naïve Bayes classifier.

The third model from which the results were collected is naïve Bayes. The performance of naïve Bayes was not on par with the other methods. The results are presented in Figure 10.

**Figure 11:**
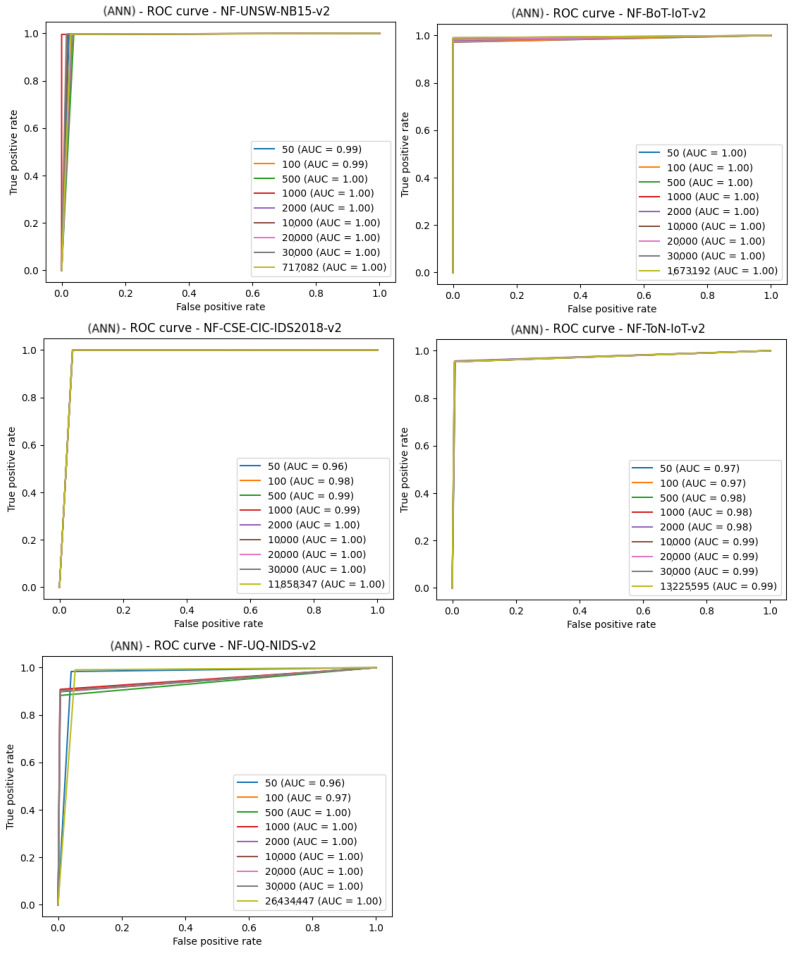
Comparison of the number of samples and machine learning effects—ROC curve plot for ANN.

The efficiency of the neural network on individual datasets is the last test conducted as part of the research contained in this paper. The neural network was built based on the configuration that was mentioned in the earlier section “Classification Models”. The entire list of studies for this set and the ANN can be found in Figure 11.

The research in this paper concludes with a comprehensive summary of the performance results of each algorithm on the five network datasets. The results of this summary can be found in Table 3. A number of metrics were used to correctly evaluate the effectiveness of a given model to verify the intrusion detection performance. It can be observed that the AdaBoost and random forest algorithms perform very well in detecting anomalies in network traffic.

## 5. Discussion

The main motivation for the research was to find the minimal set of features and the minimum size of data for intrusion detection based on the NetFlow scheme. Using five recent benchmark datasets, in the first step feature selection was performed with three methods.

The experiments showed that across all the datasets, the methods indicated common features to be the most informative. The number of selected features that do not cause a decrease in the machine learning model capability oscillates around 10 out of 33 features available in the sets. As can be observed in the figures, the importance of features drops sharply in most instances after just a few values.

The final set of features is provided by the findings coming from the use of the feature selection methods, which are presented in Figure 3, Figure 4, Figure 5, Figure 6 and Figure 7. Some features provided higher importance scores than others depending on the dataset and measuring method. However, as can be observed from the figures, many of the features are repeated, which means that these features have the greatest impact on machine learning performance, regardless of the dataset and selection method. The final list of features was compiled from the results of feature selection and compared with the results of the correlation coefficient of the features, and the features that were strongly correlated with one another were removed.

The following is the list of features extracted from the evaluated datasets using the feature selection methods. The list is validated as input data by training four different ML algorithms.

1. FLOW_DURATION_MILLISECONDS
2. TCP_WIN_MAX_IN
3. DURATION_OUT
4. MAX_TTL
5. L7_PROTO
6. SRC_TO_DST_AVG_THROUGHPUT
7. SHORTEST_FLOW_PKT
8. MIN_IP_PKT_LEN
9. TCP_WIN_MAX_OUT
10. OUT_BYTES

In the second round of research, a study was conducted on the effect of data volume on intrusion detection in network traffic. In the first stage, the available datasets were divided into smaller sets, four algorithms were trained on them, and the results were collected in the form of ROC curve plots. Following [74], the future direction is to focus on the explainability of IDS.

## 6. Conclusions and Threats to Validity

The important contribution from this study is the conclusion that large amounts of data are not needed for effective intrusion detection. The algorithms did not show much more efficiency and effectiveness after exceeding 2000 samples, which included 1000 samples of normal traffic and 1000 samples of infected traffic. Another important finding comes in the fact that not all the NetFlow fields available as features are informative features for ML classifiers.

The nature of network traffic changes over time, with new services, new equipment and novel threats all being reflected in the traffic characteristics. Thus, the datasets used for network intrusion detection have to convey those changes and the relevant, current phenomena. The rapid pace of those changes causes the datasets to become obsolete with the passage of time. This paper serves as a set of general guidelines for the collection of relevant network intrusion detection datasets adequate for stream processing solutions.

## Figures and Tables

**Table 1:** Description of the original features found in the datasets: UNSW-NB15, BoT-IoT, ToN-IoT, CSE-CIC-IDS2018, UQ-NIDS.

Feature	Description
IPV4_SRC_ADDR	IPv4 source address
IPV4_DST_ADDR	IPv4 destination address
L4_SRC_PORT	IPv4 source port number
L4_DST_PORT	IPv4 destination port number
PROTOCOL	IP protocol identifier byte
L7_PROTO	Layer 7 protocol (numeric)
IN_BYTES	Incoming number of bytes
OUT_BYTES	Outgoing number of bytes
IN_PKTS	Incoming number of packets
OUT_PKTS	Outgoing number of packets
FLOW_DURATION_MILLISECONDS	Flow duration in milliseconds
TCP_FLAGS	Cumulative of all TCP flags
CLIENT_TCP_FLAGS	Cumulative of all client TCP flags
SERVER_TCP_FLAGS	Cumulative of all server TCP flags
DURATION_IN	Client to Server stream duration (msec)
DURATION_OUT	Client to Server stream duration (msec)
MIN_TTL	Min flow TTL
MAX_TTL	Max flow TTL
LONGEST_FLOW_PKT	Longest packet (bytes) of the flow
SHORTEST_FLOW_PKT	Shortest packet (bytes) of the flow
MIN_IP_PKT_LEN	Len of the smallest flow IP packet observed
MAX_IP_PKT_LEN	Len of the largest flow IP packet observed
SRC_TO_DST_SECOND_BYTES	Src to dst Bytes/sec
DST_TO_SRC_SECOND_BYTES	Dst to src Bytes/sec
RETRANSMITTED_IN_BYTES	No. of r-d TCP flow bytes (src->dst)
RETRANSMITTED_IN_PKTS	No. of r-d TCP flow packets (src->dst)
RETRANSMITTED_OUT_BYTES	No. of r-d TCP flow bytes (dst->src)
RETRANSMITTED_OUT_PKTS	No. of r-d TCP flow packets (dst->src)
SRC_TO_DST_AVG_THROUGHPUT	Src to dst average thpt (bps)
DST_TO_SRC_AVG_THROUGHPUT	Dst to src average thpt (bps)
NUM_PKTS_UP_TO_128_BYTES	Packets whose IP size ≤ 128
NUM_PKTS_128_TO_256_BYTES	Packets whose IP size > 128 and ≤256
NUM_PKTS_256_TO_512_BYTES	Packets whose IP size > 256 and ≤512
NUM_PKTS_512_TO_1024_BYTES	Packets whose IP size > 512 and ≤1024
NUM_PKTS_1024_TO_1514_BYTES	Packets whose IP size > 1024 and ≤1514
TCP_WIN_MAX_IN	Max TCP Window (src-dst)
TCP_WIN_MAX_OUT	Max TCP Window (dst-src)
ICMP_TYPE	ICMP Type * 256 + ICMP code
ICMP_IPV4_TYPE	ICMP Type
DNS_QUERY_ID	DNS query transaction Id
DNS_QUERY_TYPE	DNS query type (e.g., 1 = A, 2 = NS.)
DNS_TTL_ANSWER	TTL of the first A record (if any)
FTP_COMMAND_RET_CODE	FTP client command return code

**Table 2:** The final result of tuning hyperparameters by using the GridSearch technique.

Model	Parameter	Value
Random Forest	n_estimators	200
	max_features	auto
	max_depth	8
	criterion	entropy
AdaBoost	n_estimators	230
	learning_rate	0.05
Naïve BAYES	var_smoothing	$10^{-9}$
ANN	epochs	16
	batch size	20
	loss function	categorical_crossentropy

**Table 3:** A summary of the effectiveness of the algorithms on each dataset using the listed metrics and established feature set.

Dataset	ACC	Precision	Recall	F1	BCC	MCC	AUC_ROC
Random Forest							
UNSW-NB15	1	1	1	1	0.9858	0.9653	0.9653
BoT-IoT	1	1	1	1	0.9989	0.9970	0.9989
CSE-CIC-IDS	1	1	1	1	0.9828	0.9800	0.9828
UQ-NIDS	0.98	0.98	0.98	0.98	0.9837	0.9557	0.9837
ToN-IoT	1	1	1	1	0.9962	0.9933	0.9962
ADABOOST							
UNSW-NB15	1	1	0.95	0.97	0.9943	0.9406	0.9943
BoT-IoT	1	1	1	1	0.9828	0.9800	0.9828
CSE-CIC-IDS	0.99	0.99	0.99	0.99	0.9666	0.9574	0.9666
UQ-NIDS	0.95	0.95	0.95	0.95	0.9540	0.8928	0.9540
ToN-IoT	0.94	0.94	0.94	0.94	0.9321	0.8690	0.9321
Naïve BAYES							
UNSW-NB15	0.98	0.98	0.98	0.98	0.8706	0.7902	0.8706
BoT-IoT	0.94	0.99	0.94	0.97	0.6275	0.0667	0.6275
CSE-CIC-IDS	0.94	0.94	0.94	0.94	0.8869	0.7303	0.8869
UQ-NIDS	0.82	0.86	0.82	0.83	0.8524	0.6644	0.8524
ToN-IoT	0.64	0.72	0.64	0.64	0.6807	0.3538	0.6807
ANN							
UNSW-NB15	1	1	1	1	0.9930	0.9164	0.9930
BoT-IoT	1	1	1	1	0.9034	0.8653	0.9034
CSE-CIC-IDS	0.99	0.99	0.99	0.99	0.9791	0.9755	0.9791
UQ-NIDS	0.97	0.97	0.97	0.97	0.9701	0.9290	0.9701
ToN-IoT	0.98	0.98	0.98	0.98	0.9681	0.9461	0.9681

## Data Availability

The data supporting the reported results are publicly available benchmarks and can be found at: https://staff.itee.uq.edu.au/marius/NIDS_datasets/ (accessed on 14 November 2021).
